# Hormonal Regulation and the Mechanistic Basis of a Sex‐Limited Color Polymorphism

**DOI:** 10.1111/ede.70072

**Published:** 2026-11-09

**Authors:** Tyra Westelius, Robin Pranter, Corin Stansfield, Natalia Zajac, Nathalie Feiner

**Affiliations:** ^1^ Department of Biology Lund University Sweden; ^2^ Max Planck Institute for Evolutionary Biology Plön Germany; ^3^ Current address: School of Biodiversity, One Health and Veterinary Medicine University of Glasgow UK

**Keywords:** Anolis lizards, color patterns, estrogen, hormone treatment, RT‐qPCR, sex reversal

## Abstract

The presence of multiple discrete color patterns within a species has captivated evolutionary biologists for more than a century, including cases in which the polymorphism is confined to one sex. The brown anole *Anolis sagrei* exhibits a female‐limited polymorphism in dorsal patterning, which is controlled by allelic variation at the autosomal *CCDC170* locus. Here, we present and test a threshold model that can explain why the polymorphism is female‐limited. We hypothesize that the hormonal environment determines whether allelic variation at the morph‐associated locus is phenotypically manifested, potentially through estrogen‐dependent regulation of *CCDC170* and its neighboring gene *ESR1*, which are more highly expressed in female than male embryos. By manipulating embryonic estradiol levels, we show that genetic males can be induced to express the polymorphism according to allelic variation at the *CCDC170* locus, which is naturally masked in males. Inversely, treating genetic females with fadrozole, which depletes estradiol, leads to monomorphic patterns irrespective of genotype, as in natural males. Using RT‐qPCR on sexual organs, we demonstrate that these effects are accompanied by changes in gene expression levels of *CCDC170* and *ESR1* in response to estradiol and fadrozole, lending support to the threshold model. Our results suggest that the *CCDC170‐ESR1‐*locus is part of a mechanistic link between the morph‐determining and the sex differentiation systems, and provide a causal explanation for the developmental origin of a sex‐limited color polymorphism.

## Research Highlights

The pattern determining locus for a female‐limited color pattern polymorphism is located on an autosome, rather than a sex chromosome. A threshold model explains how regulation through hormones links morph determination and sex differentiation.

## Graphical abstract

A threshold model explains why a color pattern polymorphism that is determined by an autosomal locus is restricted to females.

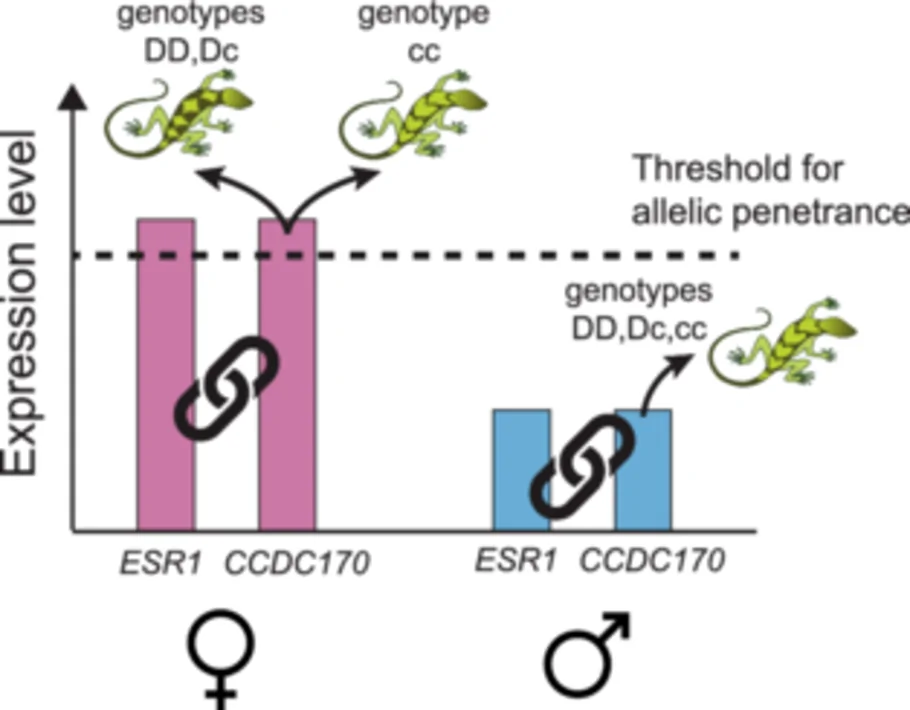

## 1 Introduction

The color patterns of fish, amphibians and reptiles arise through the spatial organization of pigment cell types (Kratochwil and Mallarino 2023; Kuriyama et al. 2020). Lizards possess three different types of pigment cells: melanophores (containing black/brown pigments), xanthophores (containing yellow/orange/red pigments), and iridophores (containing guanin crystals). These cells originate in the neural crest and migrate throughout the early embryo before settling in the integument, with the resulting color pattern largely influenced by the absolute and relative number of different types of pigment cells, their organization and cellular content. Nevertheless, the occurrence of Mendelian inherited, discrete color patterns within a species (i.e., pattern polymorphisms) suggests that transitions between morphs can be achieved by a single genetic change.

Particularly interesting cases are polymorphisms that are limited to one sex since these imply a genetic and developmental link between the generation of color pattern and the mechanism of sex differentiation (Mank 2023). For example, the morph‐determining locus can be located on the sex chromosome of the heterogametic sex, which is the case for female‐limited color polymorphism in the common cuckoo (Merondun et al. 2024) and color polymorphism in male guppies (Lindholm et al. 2004; Sandkam et al. 2021). In other cases, the morph‐determining locus has been located to an autosomal genomic region containing genes involved in sex determination, most notably *doublesex* in insects (Hendrickx et al. 2022; Nishikawa et al. 2015). However, why allelic variation located close to a sex‐determining gene can cause sex‐limited color polymorphism is poorly understood. Uncovering these mechanisms can provide insights into how changes in gene regulation contribute to the origin and evolution of color patterns.

We have previously identified the genetic basis of a female‐limited color pattern polymorphism in the brown anole lizard *Anolis sagrei* (Feiner et al. 2022), a species with genetic sex determination and well‐differentiated X and Y chromosomes (Geneva et al. 2022). Females exhibit two discrete types of dorsal color patterns, referred to as diamond and chevron, while males invariably show a chevron pattern (Figure 1A). We identified the gene *CCDC170*, encoding a protein that contains coiled‐coil domains, as a strong candidate for causing the morph differences. Females with chevron patterns carry two copies of a recessive allele we termed the c‐allele, while females with diamond patterns carry at least one copy of the dominant D‐allele, which differs from the c‐allele by 13 amino acid substitutions, likely producing a protein with a different domain structure (Feiner et al. 2022). The *CCDC170* gene in humans has been linked to cell migration and polarization (Jiang et al. 2017), and these cellular processes may be relevant for color pattern formation. Together with the extensive coding differences between the c‐ and D‐alleles, this makes functional variation in *CCDC170* a plausible mechanism underlying the morph differences. An alternative possibility, however, is that the morph‐associated haplotypes differ in regulatory elements (e.g., *cis*‐regulatory element, non‐coding RNA) located within the *CCDC170* locus, such that differences in gene regulation rather than CCDC170 protein function cause the morph differences (for such a case, see Livraghi et al. 2024). The available genetic association does not distinguish between these mechanisms.

**Figure 1:**
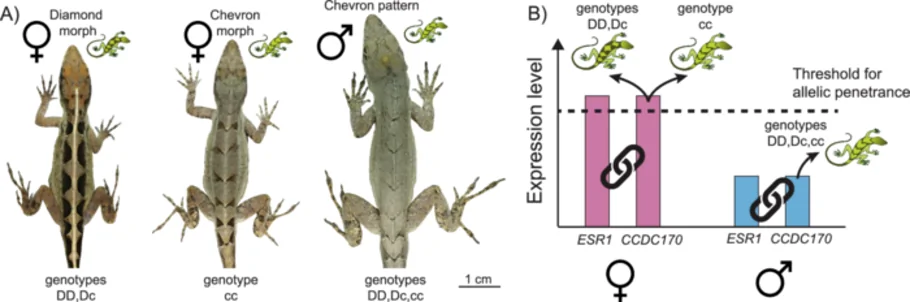
Dorsal color patterns of *Anolis sagrei* and threshold model for the effect of sex‐biased gene expression on color morph determination. (A) Representative photographs of the diamond and chevron morphs in females and the chevron pattern in males. (B) Schematic drawing of hypothetical gene expression levels of *CCDC170* and *ESR1* in females (pink bars) and males (blue bars), and illustration of a threshold for allelic penetrance below which morph determination is not affected by allelic variation at the *CCDC170* locus. In contrast, females expressing *CCDC170* above a certain threshold develop diamond patterns if at least one D‐allele is present at the *CCDC170* locus and chevron patterns if two c‐alleles are present. Chain symbols illustrate co‐expression between *CCDC170* and *ESR1*. Photograph credit: Nathalie Feiner. [Color figure can be viewed at wileyonlinelibrary.com]

The gene *CCDC170* is located on the autosomal chromosome 1, 56 kb upstream of the *Estrogen Receptor 1* gene, which plays a crucial role in female sexual development across vertebrates (Bondesson et al. 2015). The *ESR1* gene encodes the Estrogen Receptor alpha (ERα), which binds estrogen hormones such as estradiol. Estrogen binding induces a conformational change that allows ERα to act as a transcription factor and interact with estrogen‐response elements (EREs), which are DNA sequences found in promoters and other regulatory elements near target genes. Thus, in the presence of estrogen hormones, cells that express *ESR1* can switch on or off a battery of genes regulated by EREs, including *ESR1* itself.

*ESR1* shows higher expression in female embryos of *A. sagrei*, both in secondary sexual organs (hemipenes that are present in embryos of both sexes) as well as in dorsal epithelia where color pattern formation is taking place (Feiner et al. 2022). We have previously shown that the *CCDC170* gene is co‐expressed with the *ESR1* gene, potentially due to their close physical proximity, and that this co‐expression is similar for both the c‐ and D‐alleles. These observations led us to develop a threshold model that explains why the polymorphism is female‐limited. We hypothesize that low expression of *CCDC170* in males prevents allelic penetrance, effectively “masking” the pattern‐determining locus and resulting in monomorphic males (Figure 1B). In other words, an elevated expression of at least one dominant D‐allele is required to generate a diamond pattern, and this is naturally restricted to females.

If this threshold model captures the relevant dynamics correctly, experimentally increasing *ESR1* expression levels should also upregulate *CCDC170*, causing genetic males that carry a D‐allele to express the diamond morph. Similarly, experimentally decreasing *ESR1* expression levels should downregulate *CCDC170*, causing genetic females to express the chevron morph irrespective of their genotype. To test these predictions, we experimentally manipulated embryos with estradiol and fadrozole, an inhibitor of the aromatase enzyme that converts testosterone to estradiol (Bhatnagar et al. 1990). Administration of estradiol (i.e., the ERα ligand) is known to affect the expression of *ESR1* via a feedback loop. For example, studies in the Chinese soft‐shelled turtle (Chen et al. 2022), a goldfish (Marlatt et al. 2008), two snake species (Katsu et al. 2010) and a wall lizard (Cardone et al. 1998) report increased expression levels of *ESR1* upon estradiol treatment. However, both up‐ and down‐regulation of *ESR1* are reported in other species, sometimes with tissue‐specific effects (Lung et al. 2020; Shupnik et al. 1989). For example, human cell lines exposed to exogenous estradiol tend to decrease expression of *ESR1* (e.g., Saceda et al. 1988). Thus, irrespective of how estradiol treatment of embryos changes *ESR1* expression, treatment with fadrozole should have the opposite effect. Indeed, it is well‐established that estradiol and fadrozole administration during development are able to cause sex reversal in reptiles (Bull et al. 1988; Shine et al. 2007), including *A. sagrei* (Equinox 2022).

Here we investigated if administration of estradiol and fadrozole to *A. sagrei* embryos affect both sex differentiation and color morph development according to the predictions outlined above. By measuring expression levels of *ESR1* and *CCDC170*, we obtained evidence consistent with the hypothesis that co‐expression of these two genes represents a mechanistic explanation for female‐limited color polymorphism.

## 2 Materials & Methods

### 2.1 Animal Husbandry

Breeding groups of *Anolis sagrei* were kept under conditions described in (Feiner et al. 2022; Feiner et al. 2020). Animals are the 2nd and 3rd generation of a breeding stock that was set up in 2016 from adult individuals wild‐caught in Palm Coast, Florida, US. Eggs were collected every other day for two experiments as described below. In the first experiment, we administered estradiol or fadrozole to eggs to test its effect on the joint expression of sex and color pattern morph. In the second experiment, we used stressfully high and low incubation temperatures to test how incubation temperature affects variation within one morph.

### 2.2 Treatment With Estradiol or Fadrozole

Eggs were divided into two groups and each group received treatment on day 0, 5 and 10. Treatment was administered by placing a 5 µL droplet onto the porous eggshell. In 2024, the control group received 95% ethanol, while the treatment group received 0.1 µg/µL 17β‐estradiol (hereafter estradiol; for all reagents used in this study, see Supporting Information S1: Table S1) in 95% ethanol freshly prepared on the day (Ehl et al. 2017). In 2025, the control group received 95% ethanol, while the treatment group received 0.6 µg/µL fadrozole in 95% ethanol (Ehl et al. 2017). Since the estradiol and fadrozole experiments were performed in different years, we analyzed them separately (see below). Eggs were incubated at 27°C in individual small plastic containers filled two‐thirds with moist vermiculite (5:1 vermiculite:water volume ratio) and sealed with cling film. Embryos destined for RT‐qPCR were dissected at day 14 in cold nuclease‐free PBS and stored in RNA*later* (Qiagen) at −80°C. Embryos subjected to phenotyping (see below) were allowed to develop to a stage at which color patterns have formed (~28 days).

### 2.3 Manipulation of Incubation Temperature

Eggs were divided into three groups and incubated at three temperatures: low (22°C), mid (27°C) or high (32°C). According to the literature, the low and high temperatures are below and above natural nest temperatures, respectively, and may therefore cause developmental stress (Pearson and Warner 2016; Sanger et al. 2021; Sanger et al. 2018). Embryos were allowed to develop to a stage at which color patterns have formed (~70 days at low temperature, ~28 days at mid temperature and ~23 days at high temperature).

### 2.4 RT‐qPCR

Hemipenes and dorsal tissues were dissected in RNA*later* using fine needles and capsulotomy scissors and subjected to RNA extraction. Total RNA was extracted using the RNeasy Micro Kit (Qiagen) and reverse‐transcribed into cDNA using SuperScript III (Invitrogen, Carlsbad, USA) following the instructions of the 3’‐RACE System (Invitrogen, Carlsbad, USA; including RNase treatment of final cDNA). RT‐qPCRs were conducted for three genes (*GAPDH* as reference gene for internal normalization and *CCDC170* and *ESR1* as target genes) using equimolar amounts of cDNA of each sample as template and each gene/sample combination was performed in three technical replicates. For further details on methodology, including primer sequences and PCR conditions, see Feiner et al. (2022).

### 2.5 Phenotyping of Color Pattern and Anatomical Sex

Embryos that were allowed to develop to stages at which color patterns are clearly discernible were killed by transferring the eggs to −80°C. Dead embryos were dissected and pictures of their dorsal color patterns were taken. Chevron and diamond morphs show discrete differences and were scored by one observer (T.W.). Variation within the chevron morph was not quantified but we scored variation within the diamond morph that can be described as a continuum from a single longitudinal bar to a diamond pattern. We captured this variation using a sinuosity score (cf. Moon and Kamath 2019) that is calculated as the geometric mean of the length of left and right diamond outline divided by the length of the midline of the dorsum between fore‐ and hind‐limb insertions. A sinuosity score close to 1 indicates a bar‐like pattern while an increasing score indicates a more diamond‐like pattern. Anatomical sex was scored based on presence or absence of hemipenes.

### 2.6 Genotyping and Molecular Sexing

Tail tissue was collected from each embryo and DNA was extracted using the DNeasy Blood & Tissue Kit (Qiagen, USA). We used two PCR‐based genotyping assays to determine the *CCDC170* genotype and to determine the genetic sex following methods described in Feiner et al. (2022). In brief, the *CCDC170* genotype was determined by amplifying a 710‐bp fragment of exon 2 and treating the PCR products with the restriction enzyme Taq I, whose recognition site is present in the D‐, but not the c‐allele. For molecular sexing, we used the primers AcarB previously developed by Gamble and Zarkower (2014), which target a male‐specific fragment of 238 bp located on the Y chromosome (for details, see Feiner et al. 2022).

### 2.7 Statistical Analyses

We conducted all statistical analyses in R (version 4.5.2; R Core Team, 2017). Differences in embryo survival, deviations from 1:1 sex ratios, and differences in the frequencies of morphs correctly predicted from genotypes were assessed using Fisher's exact tests and *χ* ^2^ tests, as appropriate. To assess the effects of treatment, sex, and their interaction on gene expression levels, we fitted linear models. When interaction terms were not significant *(p* > 0.3), they were removed and main effects were interpreted from the reduced model. When a significant interaction was detected, we conducted *post hoc* analyses using estimated marginal means implemented in the emmeans package (version 2.0.1), with pairwise comparisons to evaluate treatment effects within each sex. Differences in sinuosity scores were analyzed using Analysis of Variance, followed by *post hoc* pairwise comparisons using Tukey's HSD.

### 2.8 Genomic Characterization of the CCDC170‐ESR1 Locus

To predict estrogen‐response elements (EREs) in the vicinity of the *CCDC170‐ESR1* genomic region and across the entire *Anolis sagrei* genome, we downloaded the consensus motif from Jaspar (https://jaspar.elixir.no/; accessed 29.01.2026; Matrix ID: MA0112.1). The DNA‐binding domains of ERα receptors are identical between human, chicken, and alligator (Rice et al. 2017), suggesting a high level of conservation of ERα, and thus EREs, among tetrapods. We therefore performed motif discovery in the *A. sagrei* genome (GCF_037176765.1) with this consensus motif using the command line version of FIMO v5.5.9 from MEME‐SUITE. A statistical threshold of *p* < 0.01 was used for discovery of motif occurrence.

To assess if the *CCDC170* and *ESR1* genes are located in tandem also in other vertebrate taxa, we used OMA (https://omabrowser.org/) to examine the genomic neighborhood of the *CCDC170* gene across 139 vertebrate species belonging to the Hierarchical Orthologous Group HOG:E0789397.1a (accessed March 2026). Local gene‐centric synteny in OMA indicated this collinearity is highly conserved across all vertebrates except in actinopterygian fish. We thus selected representative species per clade and conducted a collinearity analysis of the broader genomic region (+/− 2 Mb of the focal gene) in 23 vertebrate species. Annotations were downloaded from NCBI RefSeq (Supporting information S1: Table S2). Regions in the genome containing *CCDC170* together with neighboring genes were visualized in R using the R package gggenomes (v1.0.1).

## 3 Results

### 3.1 Treating Developing Embryos With Estradiol and Fadrozole Affects Sexual Development

A total of 88 and 41 eggs were treated with estradiol and fadrozole, respectively, or a control treatment using 95% ethanol (control sample size: estradiol experiment, 69; fadrozole experiment, 35). Embryo survival was above 94% across all four treatment groups and did not differ between treatment and control groups (estradiol treated, 94.3%; estradiol control, 98.6%; Fisher's exact test, *p* = 0.23; fadrozole treated, 95.1%; fadrozole control, 94.3%; *p* = 1).

As previously reported in this and other species (Ehl et al. 2017; Equinox 2022), the anatomical sex was affected by treatment with estradiol or fadrozole. At an embryonic stage close to hatching, males typically possess hemipenes that are clearly visible as paired structures (Figure 2A). While hemipenes are also present in females at earlier stages of development, they have regressed and are not visible at the embryonic stage assessed here (Figure 2A). Treating developing eggs with estradiol caused all embryos (*N* = 83) to develop into anatomical females that lack visible hemipenes, while treatment with fadrozole caused all embryos (*N* = 39) to develop into anatomical males with hemipenes (Figure 2B). Molecular sexing verified that all four treatment groups showed the expected 1‐to‐1 genetic sex ratio (estradiol experiment, *χ* ^2^_(1)_ = 0, *p* = 1; fadrozole experiment, *χ* ^2^_(1)_ = 0.07, *p* = 0.80). Individuals were not raised to sexual maturity and it is therefore unknown to what extent primary sexual organs and sexual behavior are affected.

**Figure 2:**
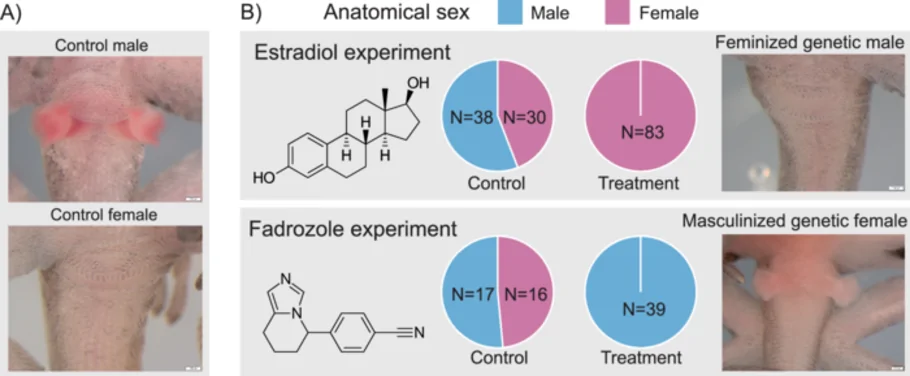
Effects of estradiol and fadrozole on anatomical sex. (A) In control animals that were treated with ethanol, genetic males exhibit clearly visible hemipenes, while genetic females lack these structures at late embryonic stages. (B) This characterization of the anatomical sex is affected by administration of estradiol (feminizing effect) or fadrozole (masculinizing effect). Pie charts present the number of anatomical males and females per treatment group (control or treatment) and experiment (estradiol or fadrozole). Images to the right show examples of the cloacal region of a feminized genetic male (estradiol treatment) and a masculinized genetic female (fadrozole treatment). [Color figure can be viewed at wileyonlinelibrary.com]

### 3.2 Treatment With Estradiol Leads to Upregulation of *ESR1* Expression Via a Positive Feedback Loop

The consistent effect of estradiol and fadrozole on the anatomical sex before hatching may be due to their effect on the expression of the estradiol receptor, *ESR1*. Characterizing the genomic region of *ESR1*, we found that there are eight EREs in its vicinity (Figure 3A), which is more than was observed for other genes in the *A. sagrei* genome (*p* = 0.040). Three of these EREs are directly upstream of the first exon. In addition, there were two EREs located downstream of *ESR1*, and two in the gene *CCDC170*, upstream of *ESR1*. This makes it likely that transcriptional regulation of *ESR1* is regulated by estradiol via a positive feedback loop, as has been shown for other vertebrates (see Introduction).

**Figure 3:**
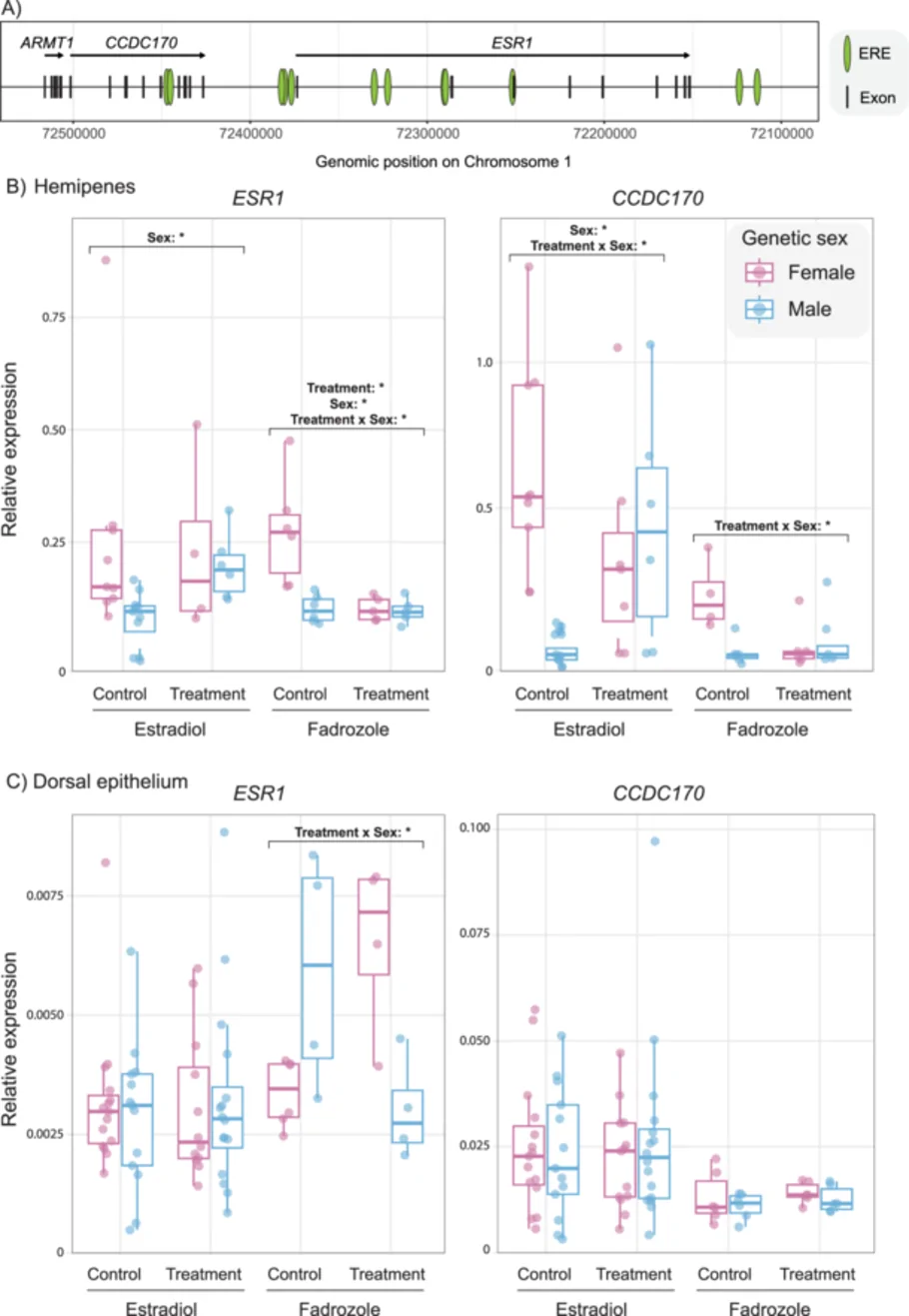
Effect of estradiol and fadrozole on the expression levels of *ESR1* and *CCDC170*. (A) Predicted location of EREs in the genomic environment of *CCDC170* and *ESR1* are shown as green ellipses. (B and C) Relative expression of the two target genes (normalized using *GAPDH*) are plotted for hemipenes (panel B) and dorsal epithelia (panel C). Each datapoint presents the mean of three technical replicates. Boxes show interquartile ranges with middle line showing the median. Whiskers extend to 1.5 times the interquartile ranges. Significant effects are highlighted with brackets (see also Table 1). [Color figure can be viewed at wileyonlinelibrary.com]

For each treatment group, we performed RT‐qPCR on hemipenes of embryos at embryonic stages 11–12 (staging based on Sanger et al. 2008) to quantify expression levels of *ESR1*. At this stage, both sexes possess hemipenes, but they are larger in males. Genetic sex had an overall significant effect on expression levels of *ESR1* in hemipenes, while the effect of treatment was negligible (Table 1 and Figure 3B). A *post hoc* test revealed that females in the control group showed a higher *ESR1* expression level in hemipenes than males in the control group (*t* = 2.339, df = 26, *p* = 0.027), but that this difference disappeared when embryos were treated with estradiol (*t* = 0.331, df = 26, *p* = 0.744).

**Table 1:** Effect of treatment of developing embryos with estradiol and fadrozole on expression levels of ESR1 and CCDC170 in hemipenes and dorsal epithelia.

Tissue	Gene	Experiment	Treatment	Sex	Treatment x Sex
Hemipenes	*ESR1*	Estradiol	*F* = 0.610	***F*** = **4.445**	*F* = 1.134
			*p* = 0.442	***P*** = **0.045**	*p* = 0.297
		Fadrozole	***F*** = **9.290**	***F*** = **11.088**	***F*** = **8.365**
			***P*** = **0.007**	***P*** = **0.004**	***P*** = **0.010**
	*CCDC170*	Estradiol	*F* = 1.109	***F*** = **10.370**	***F*** = **10.875**
			*p* = 0.300	***P*** = **0.003**	***P*** = **0.002**
		Fadrozole	*F* = 0.838	*F* = 3.119	***F*** = **6.829**
			*p* = 0.373	*p* = 0.095	***P*** = **0.018**
Dorsal epithelium	*ESR1*	Estradiol	*F* = 0.045	*F* = 0.022	*F* = 0.254
			*p* = 0.833	*p* = 0.882	*p* = 0.616
		Fadrozole	*F* = 0.258	*F* = 0.077	***F*** = **16.165**
			*p* = 0.620	*p* = 0.785	***P*** = **0.001** *
	*CCDC170*	Estradiol	*F* = 0.012	*F* = 0.153	*F* = 0.419
			*p* = 0.912	*p* = 0.697	*p* = 0.520
		Fadrozole	*F* = 0.711	*F* = 1.192	*F* = 0.025
			*p* = 0.409	*p* = 0.288	*p* = 0.875

*Note:* Significant effects are highlighted in bold.

* Note that although the interaction term is significant, a *post hoc* test shows that the sex‐biased gene expression in *ESR1* in dorsal epithelia is not erased by treatment with fadrozole, but is reversed for the sexes (fadrozole‐treated: *t* = 3.157, df = 14, *p* = 0.007; control: *t* = −2.506, df = 14, *p* = 0.025).

Treatment with fadrozole showed a similar effect in terms of removing sex‐biased gene expression: while both treatment and genetic sex had a significant effect on the expression of *ESR1* in hemipenes (Table 1 and Figure 3B), a *post hoc* test showed that the difference between the sexes observed in the control groups disappears upon treatment with fadrozole (fadrozole‐treated: *t* = 0.109, df = 18, *p* = 0.915; control: *t* = 4.409, df = 18, *p* < 0.001). Overall, these results indicate that treatment with estradiol upregulates the naturally low *ESR1* expression in male hemipenes to levels typical of females, while fadrozole downregulates the naturally high *ESR1* expression in female hemipenes to levels typical of males (Figure 3B).

Consistent with previous findings reporting co‐expression between *ESR1* and the neighboring gene *CCDC170* (Feiner et al. 2022), we found that *CCDC170* expression levels respond in similar ways as *ESR1* in hemipenes upon estradiol and fadrozole treatment (Figure 3C). While neither treatment had a significant main effect (Table 1 and Figure 3C), *post hoc* tests demonstrated that estradiol increases *CCDC170* expression in treated genetic males to levels typical of genetic females (estradiol‐treated: *t* = −0.621, df = 31, *p* = 0.539; control: *t* = 4.567, df = 31, *p* < 0.001). *Vice versa*, fadrozole‐treatment reduced the *CCDC170* expression in treated genetic females to levels typical of genetic males (fadrozole‐treated: *t* = −0.500, df = 17, *p* = 0.624; control: *t* = 3.114, df = 17, *p* = 0.006; Figure 3C).

To test if the strong, sex‐specific effect of estradiol and fadrozole on *ESR1* and *CCDC170* in developing hemipenes is also observed in dorsal epithelia, the tissue in which color patterns are formed, we performed the equivalent RT‐qPCRs on dissected dorsal epithelia. We found that the overall, mean expression levels of *ESR1* and *CCDC170* were more than a magnitude lower in dorsal epithelia relative to hemipenes (mean expression levels: *ESR1*_Hemipenes_ = 0.165; *ESR1*_Dorsal Epithelium_ = 0.003; *CCDC170*_Hemipenes_ = 0.242; *CCDC170*_Dorsal Epithelium_ = 0.021). The only significant effect of sex and treatment was found for fadrozole, which appears to reverse sex‐biased expression of *ESR1* (Table 1).

Overall, we conclude that administration of estradiol leads to upregulation of *CCDC170* and *ESR1* via a positive feedback loop, while fadrozole has the opposite effect. However, the estradiol treatment appears to have limited quantitative effect on the naturally high expression of these genes in females, and, similarly, the fadrozole treatment seems to have limited effect on the naturally low expression in males. This results in an absence of sex‐biased gene expression of *CCDC170* and *ESR1* in estradiol‐ and fadrozole‐treated embryos.

### 3.3 Treatment With Estradiol and Fadrozole Affects Morph Determination in a Predictable Way

Given that estradiol treatment increases, and fadrozole treatment decreases, *CCDC170* (and *ESR1*) expression levels, the threshold model predicts corresponding effects on the expression of color pattern morphs. Specifically, experimentally increasing *CCDC170* levels in genetic males using estradiol should ‘unmask’ the effects of allelic variation (c‐ or D‐alleles) on color morph determination. Conversely, experimentally decreasing *CCDC170* levels in genetic females using fadrozole should “mask” allelic variation at this morph determining locus, leading to the expression of the chevron morph, regardless of the *CCDC170* genotype.

In line with these predictions, we observed that all genetic males in the control treatment expressed the chevron color pattern, while treating genetic males with estradiol caused them to develop color patterns according to their *CCDC170* genotypes: all 34 estradiol‐treated genetic males with DD or Dc genotypes developed a diamond morph, while 9 out of 10 estradiol‐treated males with the cc genotype showed a chevron pattern (Figure 4A). Thus, 43 out of 44 estradiol‐treated genetic males developed a color morph that was predicted by their *CCDC170* genotypes, while this was only the case for 10 out of 36 animals in the control group where all were chevron morphs (Fisher's exact test, *p* < 0.001). In comparison, fadrozole treatment had no effect on color morph in genetic males, with all embryos of both treatment and control groups expressing the chevron morph, regardless of their underlying *CCDC170* genotypes (Fisher's exact test, *p* = 0.125).

**Figure 4:**
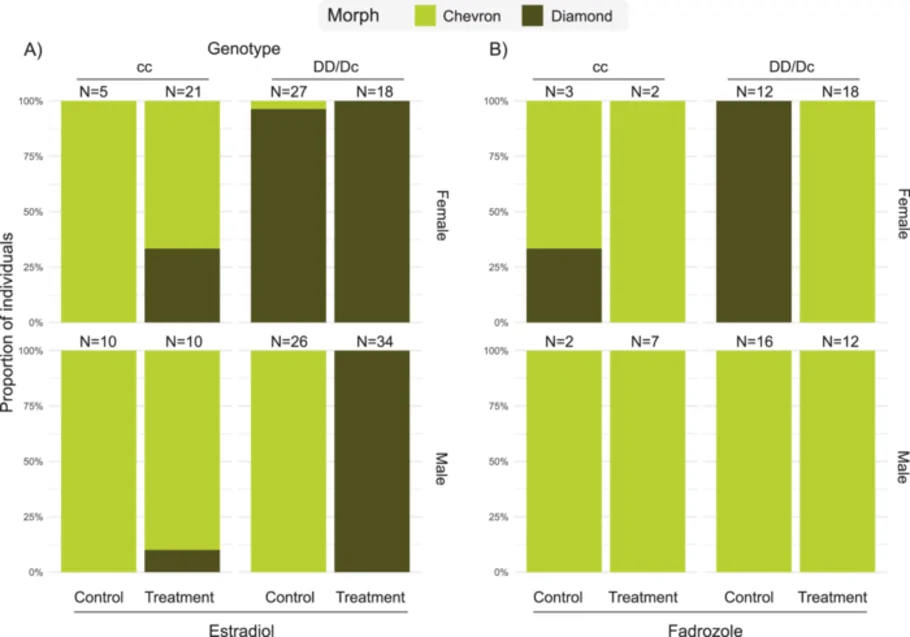
Effect of estradiol and fadrozole on color pattern morph in *A. sagrei*. (A) Bar plots show the numbers of individuals showing chevron (green) and diamond (brown) morph for genetic females and males in the estradiol experiment treated either with estradiol (treatment) or ethanol (control). (B) Same as in panel A, but for the fadrozole experiment. [Color figure can be viewed at wileyonlinelibrary.com]

Conversely, treating genetic females with estradiol had no effect on the relationship between *CCDC170* genotypes and color morph, and genotype predicted the observed color morph in 31 out of 32 cases in the control group and 32 out of 39 cases in the estradiol‐treatment group (Fisher's exact test, *p* = 0.065). Fadrozole treatment on the other hand, had a significant impact: all 18 genetic females that had a DD or Dc genotype treated with fadrozole developed a chevron phenotype (Figure 4B). While *CCDC170* genotypes predicted the correct color morph in 14 out of 15 cases in the control group in genetic females, this was only the case for 2 out of 20 individuals in the fadrozole treatment group (Fisher's exact test, *p* < 0.001). Thus, estradiol treatment “unmasked” the effect of the *CCDC170* genotype on morph determination in genetic males, while fadrozole “masked” the effect in females. These findings are in agreement with the threshold model and suggest that the expression of *CCDC170* determines whether or not the allelic variation at this locus is manifested as color pattern morph determination.

### 3.4 Effects of Sex and Environment on Quantitative Aspects of the Diamond Pattern

After establishing that genetic males with at least one ‘D’ allele at the *CCDC170* locus develop a diamond pattern when treated with estradiol, we investigated whether these diamond patterns differ quantitatively from the naturally observed diamond patterns of females. We found that the sinuosity score was significantly different between males treated with estradiol and females treated with estradiol or control (*F* = 6.64; *p* = 0.002). A *post hoc* test showed that color patterns of males treated with estradiol are significantly more bar‐like compared to females of the control treatment (*p* = 0.002). Estradiol‐treated females also showed a trend towards a more bar‐like pattern but this difference did not reach statistical significance (*p* = 0.128; Figure 5A,B).

**Figure 5:**
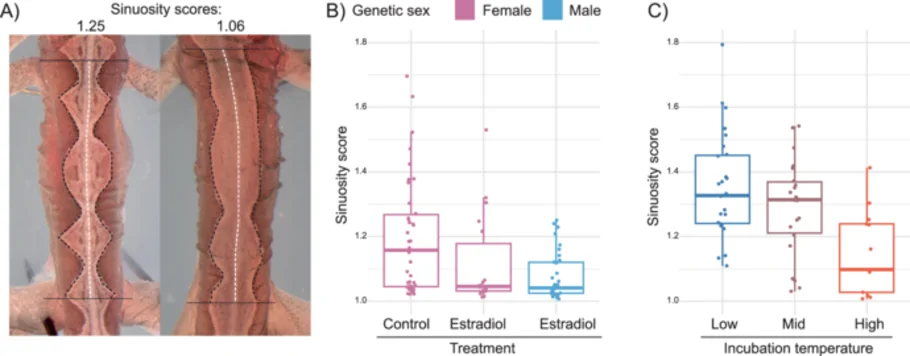
Effect of estradiol and incubation temperature on diamond pattern formation. (A) Two diamond patterns exemplifying a diamond‐like (left) and a more bar‐like (right) phenotype. The sinuosity score is derived from the geometric mean of the length of left and right diamond outline (black dashed lines) divided by the length of the midline (white dashed line) between fore‐ and hind‐limb insertions (marked by grey horizontal lines). (B) Sinuosity scores of genetic females treated with ethanol (control; *N* = 38) or estradiol (*N* = 18) and genetic males treated with estradiol (*N* = 34). (C) Sinuosity scores of untreated diamond‐morph females that were incubated at low (*N* = 31), mid (*N* = 27) or high (*N* = 14) temperatures. Boxes show interquartile ranges with middle line showing the median. Whiskers extend to 1.5 times the interquartile ranges. [Color figure can be viewed at wileyonlinelibrary.com]

To test if this quantitative variation within the diamond morph reflects a disruption of optimal developmental conditions, we conducted an experiment in which we exposed developing embryos to stressfully low and high, in addition to optimal (here referred to as “mid”) incubation temperatures. In particular high incubation temperatures have been shown to lead to developmental stress (Sanger et al. 2021; Sanger et al. 2018). We found significant differences between the three temperature groups (*F* = 8.22; *p* < 0.001), with the high temperatures causing significantly more bar‐like patterns with low sinuosity scores (low‐high, *p* < 0.001; mid‐high, *p* = 0.027; Figure 5C). This suggests that high temperatures as well as hormone levels quantitatively influence dorsal pattern formation in *A. sagrei*, but that this effect does not ‘override’ the effect of D‐ and c‐alleles on morph determination.

### 3.5 Coupling Between Sex‐ and Morph‐Determination at the Genomic Level

Our results suggest that morph determination is linked to sex differentiation through the genetic linkage between *CCDC170* and *ESR1*. Given that female‐limited polymorphisms have been reported in other vertebrates (Dijkstra et al. 2007; Falk et al. 2022), we investigated if the collinearity between the two genes is broadly conserved. We find that across vertebrates, *CCDC170* and *ESR1* were located next to each other and in the same orientation in all ten investigated mammals, in all six reptiles (incl. birds), in both amphibians and in all three chondrichthyans (Figure 6). Within actinopterygian fish, the early‐branching spotted gar (*Lepisosteus oculatus*) preserved the linkage between *CCDC170* and *ESR1*, while the two genes are located on different chromosomes in the two teleost fish that we investigated. The overall high level of conservation in collinearity may indicate that the mechanisms for sex‐linked color pattern morphs in *Anolis sagrei* may be broadly applicable to lizards and other vertebrates.

**Figure 6:**
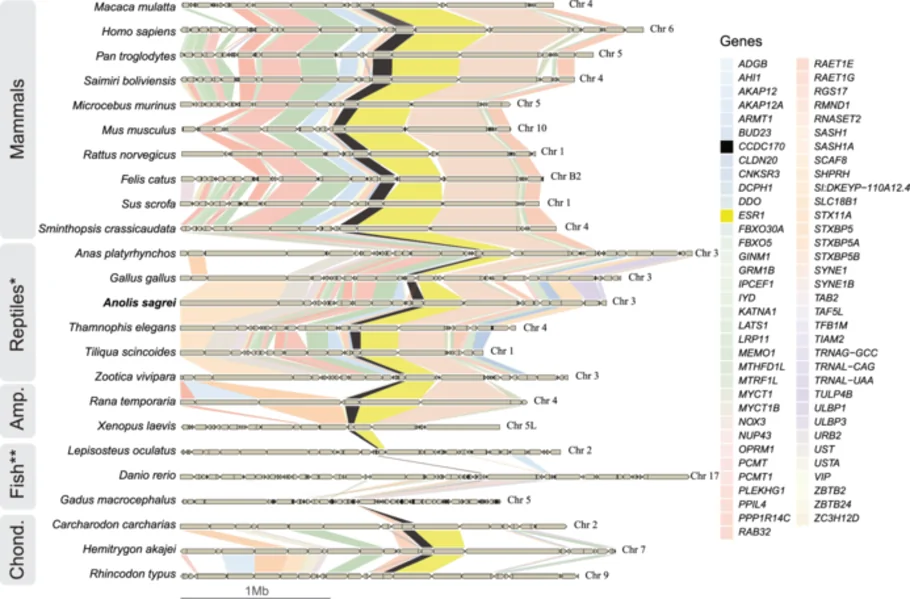
Conservation of the *CCDC170‐ESR1*‐locus in vertebrate genomes. Homology of genes in the *CCDC170‐ESR1* surrounding region is shown by colored rectangles, and the direction of transcription is indicated by a triangle at the 3′‐end. Intergenic regions are omitted from this figure. The focal species, *Anolis sagrei*, is shown in bold, and *CCDC170* is marked in black and *ESR1* in yellow. Representative species were selected from mammals, reptiles (* including birds), amphibians, fish (** actinopterygian fish) and chondrichthyans. [Color figure can be viewed at wileyonlinelibrary.com]

## 4 Discussion

The female‐limited color pattern polymorphism in *Anolis sagrei* has been mapped to the *CCDC170* locus, which is located directly upstream of the *ESR1* gene that is more highly expressed in female than in male embryos (Feiner et al. 2022). This genomic configuration provides a potential explanation for why the morph is sex‐limited, and we propose a threshold model that captures why allelic variation at the color determining locus is masked in males in an estrogen‐dependent manner. In support of this model, we show that genetic male embryos treated with estradiol, causing them to express *ESR1* and *CCDC170* at levels similar to those of genetic females, developed color morphs in accordance with allelic variation at the *CCDC170* locus. Similarly, reducing expression of *ESR1* and *CCDC170* with fadrozole caused genetic females to develop a chevron morph even if they carry alleles associated with the diamond morph. These results demonstrate that the hormonal environment determines whether allelic variation at the morph‐associated locus is phenotypically manifested. However, they do not resolve whether the underlying morph differences are caused by functional variation in the CCDC170 protein or by regulatory variation associated with this locus.

What is the molecular mechanism by which allelic variation at this locus affects morph determination? One possibility is that the *CCDC170* gene itself is the causal factor. This hypothesis is supported by the 13 amino acid substitutions distinguishing the morph‐associated c‐ and D‐alleles, which are predicted to result in proteins with different domain structures (Feiner et al. 2022), and by the reported role of CCDC170 in cell migration and polarization (Jiang et al. 2017). Under this model, sex‐biased expression of *CCDC170* determines whether functional differences between the c‐ and D‐alleles become phenotypically penetrant. *CCDC170* could be directly regulated by estradiol via the two predicted estrogen‐response elements (EREs) located in an intron of *CCDC170*. Alternatively, its co‐expression with *ESR1*, which has been documented in humans (Dunbier et al. 2011) and in *A. sagrei* (Feiner et al. 2022), could result from their physical proximity or a shared regulatory landscape with common epigenetic features (e.g., DNA methylation or histone modifications). *CCDC170* could also be regulated through an unknown factor in trans that is itself regulated by estradiol.

An alternative hypothesis is that the morph‐associated haplotypes may contain different regulatory elements, such that differences in gene regulation rather than CCDC170 protein function cause the morph differences. The potential target gene or genes of such regulatory variation are currently unknown. *ESR1* represents one plausible target because of its close physical proximity and its role in estrogen signaling. The fact that administration of estradiol and fadrozole leads to sex reversal demonstrates the pervasive, systemic effects of these treatments and suggests that expression levels of many genes are estradiol‐sensitive. Consequently, the hormone manipulations used here cannot identify which estradiol‐responsive gene ultimately mediates the effect on morph determination. Our current data cannot distinguish between these two hypotheses. While the extensive coding differences between the morph‐associated *CCDC170* alleles provide support for *CCDC170* as a candidate effector, we currently have no evidence identifying a morph‐associated regulatory variant or its target gene. Discriminating between these mechanisms will require direct functional interrogation of the regulatory landscape of this locus or independent manipulation of *CCDC170*, *ESR1*, and candidate regulatory elements.

Taking an evolutionary perspective, an intriguing question is whether this genomic configuration in which the morph‐associated *CCDC170* locus is located adjacent to a gene crucial for sex differentiation has been exploited by other lineages. To the best of our knowledge, in all vertebrate cases for which the genetic basis of a female‐limited polymorphism has been resolved, the morph determining locus has been located on a sex chromosome (para guppy, Lindholm et al. 2004; common cuckoo, Merondun et al. 2024; Sandkam et al. 2021). Yet, the deep evolutionary conservation of the collinearity of *CCDC170* and *ESR1* across vertebrates (with the exception of teleost fish) raises the possibility that involvement of the *CCDC170‐ESR1*‐locus in the evolution of female‐limited color pattern polymorphisms may be more widespread. Investigating the genetic basis of such polymorphisms in other *Anolis* species (Medina et al. 2016) and other vertebrates, such as the white‐necked jacobin hummingbird (Falk et al. 2022) may reveal further instances. Taking a comparative approach to reconstruct the evolutionary steps that led up to the genetic architecture integrating morph determination and sex differentiation would provide insights into the mechanistic underpinnings of evolutionary transitions between sex‐limited and universal, and between mono‐ and polymorphic color pattern systems.

A remaining question is how allelic variation at this locus affects the migration, differentiation or other behaviors of pigment cells that generate color patterns. In contrast to our previous work (Feiner et al. 2022), we did not detect consistent effects of treatment or sex on the expression of *CCDC170* in the tissue where pattern formation is taking place, the dorsal epithelia. Possible explanations may be that the experiment reported by Feiner et al. (2022) and those reported here used slightly different developmental stages or technical variation in the micro‐dissection of the epithelial tissues, which resulted in overall lower expression levels in the present study. Pigment cells are embedded in various other cell types, which means that the extent to which other tissue layers are included could affect variation in qPCR results between studies. Application of single‐cell RNA‐sequencing or spatial transcriptomic techniques could overcome this problem by generating *CCDC170* and *ESR1* expression levels by cell type and could also help identify other genes whose expression covaries with morph‐associated genotype in the relevant cell populations. Such approaches would therefore be informative for both the *CCDC170*‐dependent and regulatory hypotheses.

Our work demonstrates that morph determination is genetically controlled and sensitive to hormones, and, in addition, we also obtained important insights into the regulation of within‐morph variation. Prompted by the observation that estradiol‐treated males develop diamond morphs that are significantly more bar‐like relative to those observed in control (ethanol‐treated) females, we tested if also incubation temperature affects the quality of the expressed diamond pattern. Incubation temperatures outside natural ranges, in particular higher temperature (Angilletta 2009; Pettersen et al. 2023), are known to cause developmental stress that can manifests in pervasive changes in global transcriptomic profiles (Feiner et al. 2018) as well as in specific gene expression changes associated with specific phenotypes, for example a shortened snout (Sanger et al. 2021). We found that stressfully high incubation temperatures gave rise to more bar‐like diamond patterns relative to low or optimal temperatures. Together, these observations suggest that bar‐like patterns may be a sign of developmental stress caused by high incubation temperatures or unnaturally high levels of estradiol. In line with this interpretation, we observed that ethanol‐treated (control) females tended to show more bar‐like patterns relative to untreated females incubated at optimal incubation temperatures (mid ranges in temperature incubation experiment), which leads us to speculate that not only high incubation temperature and unnaturally high levels of estradiol, but also ethanol may cause developmental stress. Unfortunately, our current understanding of the cellular developmental process of diamond pattern formation, and pattern formation more generally, remains too limited to allow any speculation on how this stress is translated into cellular behaviors that manifest in bar‐like rather than diamond‐like patterns.

## 5 Conclusion

Here, we present and experimentally validate a threshold model that captures the cross‐talk between the color morph determination and sex differentiation systems to explain the female‐limited diamond‐chevron polymorphism in the brown anole lizard *Anolis sagrei*. Our results demonstrate that the hormonal environment determines whether allelic variation at the morph‐associated *CCDC170‐ESR1* locus is phenotypically manifested, thereby providing a mechanistic link between morph determination and sex differentiation. Whether this coupling is mediated by functional variation in the CCDC170 protein or by regulatory variation associated with this locus remains to be resolved. Together, our findings provide insights into the developmental origins of a sex‐limited color polymorphism.

## Ethics Statement

The study was conducted according to the Lund University Local Ethical Review Process, the Federation of European Laboratory Animal Science Associations guidelines and the German animal welfare law (Tierschutzgesetz § 11).

## Conflicts of Interest

The authors declare no conflicts of interest.

## Supporting information


Supporting File

## Data Availability

Data and code that was used to generate the results presented in this study are available at Zenodo: (URL: https://zenodo.org/records/23685387).
