# Supplementary material for: Hormonal Regulation and the Mechanistic Basis of a Sex‐Limited Color Polymorphism
Source: Evol Dev. 2026 Nov 9;28(4):e70072. doi: 10.1111/ede.70072 (PMC13747244; doi:10.1111/ede.70072)
Supplement: Supplementary file 1 [file EDE-28-e70072-s001.pdf]

Supplementary material

Hormonal regulation and the mechanistic basis of a sex-limited color polymorphism

Tyra Westelius[1], Robin Pranter[1,2], Corin Stansfield[1,3], Natalia Zajac[2] and Nathalie Feiner[1,2]

[1]Department of Biology, Lund University, Sweden

[2]Max Planck Institute for Evolutionary Biology, Plön, Germany

[3]Current address: School of Biodiversity, One Health and Veterinary medicine, University of Glasgow, United Kingdom

This document contains 2 Supplementary Tables.

Supplementary material

Supplementary Table S1. List of reagents used in this study.

Reagent	Company	Catalog#	Cas#
DNeasy Blood & Tissue Kit	Qiagen	69504	
GoTaq Hot Start Master Mix	Promega	M5122	
TaqI-v2	New England Biolabs	R0149S	
rCutSmart Buffer	New England Biolabs	B6004S	
RNeasy Micro Kit	Qiagen	74004	
5x First strand buffer	Invitrogen	Y02321	
Dithiothreitol (DTT)	Invitrogen	Y00147	
SupersScript III reverse transcriptase	Invitrogen	18080	
RNasin Plus RNase Inhibitor	Promega	N261A	
RNase H	New England Biolabs	M0297S	
Platinum SYBR Green qPCR SuperMix-UDG	Thermo Fisher Scientific	11733046	
ROX	Invitrogen	54881	50-28-2
Phosphate buffered saline (PBS)	Sigma-Aldrich	P4417	7732-18-5
Water, nucl. free, Mol. Biol. Grade, Ultrapure	ThermoScientific	J71786.K8	
17β-Estradiol	Cayman chemical	10006315	102676-31-3
Fadrozole hydrochloride	Sigma-Aldrich	F3806	
RNAlater Solution	Invitrogen	AM70	

Supplementary material

Supplementary Table S2. Genomes and genome annotations used for collinearity analysis.

File	Species	FTP	Accession
GCF_000001635.27_GRCm39_genomic.gff	*Mus musculus*	https://ftp.ncbi.nlm.nih.gov/genomes/all/GCF/000/001/635/GCF_000001635.27_GRCm39/	GCF_000001635.27
GCF_000003025.6_Sscrofa11.1_genomic.gff	*Sus scrofa*	https://ftp.ncbi.nlm.nih.gov/genomes/all/GCF/000/003/025/GCF_000003025.6_Sscrofa11.1/	GCF_000003025.6
GCF_018350175.1_F.catus_Fca126_mat1.0_genomic.gff	*Felis catus*	https://ftp.ncbi.nlm.nih.gov/genomes/all/GCF/018/350/175/GCF_018350175.1_F.catus_Fca126_mat1.0/	GCF_018350175.1
GCF_035046505.1_rTilSci1.hap2_genomic.gff	*Tiliqua scincoides*	https://ftp.ncbi.nlm.nih.gov/genomes/all/GCF/035/046/505/GCF_035046505.1_rTilSci1.hap2/	GCF_035046505.1
GCF_009914755.1_T2T-CHM13v2.0_genomic.gff	*Homo sapiens*	https://ftp.ncbi.nlm.nih.gov/genomes/all/GCF/009/914/755/GCF_009914755.1_T2T-CHM13v2.0/	GCF_009914755.1
GCF_016699485.2_bGalGal1.mat.broiler.GRCg7b_genomic.gff	*Gallus gallus*	https://ftp.ncbi.nlm.nih.gov/genomes/all/GCF/016/699/485/GCF_016699485.2_bGalGal1.mat.broiler.GRCg7b/	GCF_016699485.2
GCF_017639515.1_sCarCar2.pri_genomic.gff	*Carcharodon carcharias*	https://ftp.ncbi.nlm.nih.gov/genomes/all/GCF/017/639/515/GCF_017639515.1_sCarCar2.pri/	GCF_017639515.1

GCF_017654675.1_Xenopus_laevis_v10.1_genomic.gff	*Xenopus laevis*	https://ftp.ncbi.nlm.nih.gov/genomes/all/GCF/017/654/675/GCF_017654675.1_Xenopus_laevis_v10.1/	GCF_017654675.1
GCF_028858775.2_NHGRI_mPanTro3-v2.0_pri_genomic.gff	*Pan troglodytes*	https://ftp.ncbi.nlm.nih.gov/genomes/all/GCF/028/858/775/GCF_028858775.2_NHGRI_mPanTro3-v2.0_pri/	GCF_028858775.2
GCF_031168955.1_ASM3116895v1_genomic.gff	*Gadus macrocephalus*	https://ftp.ncbi.nlm.nih.gov/genomes/all/GCF/031/168/955/GCF_031168955.1_ASM3116895v1/	GCF_031168955.1
GCF_036323735.1_GRCr8_genomic.gff	*Rattus norvegicus*	https://ftp.ncbi.nlm.nih.gov/genomes/all/GCF/036/323/735/GCF_036323735.1_GRCr8/	GCF_036323735.1
GCF_037176765.1_rAnoSag1.mat_genomic.gff	*Anolis sagrei*	https://ftp.ncbi.nlm.nih.gov/genomes/all/GCF/037/176/765/GCF_037176765.1_rAnoSag1.mat/	GCF_037176765.1
GCF_040939455.1_M.murinus_Inina_mat1.0_genomic.gff	*Microcebus murinus*	https://ftp.ncbi.nlm.nih.gov/genomes/all/GCF/040/939/455/GCF_040939455.1_M.murinus_Inina_mat1.0/	GCF_040939455.1
GCF_047663525.1_IASCAAS_PekinDuck_T2T_genomic.gff	*Anas platyrhynchos*	https://ftp.ncbi.nlm.nih.gov/genomes/all/GCF/047/663/525/GCF_047663525.1_IASCAAS_PekinDuck_T2T/	GCF_047663525.1
GCF_048418815.1_sHemAka1.3_genomic.gff	*Hemitrygon akajei*	https://ftp.ncbi.nlm.nih.gov/genomes/all/GCF/048/418/815/GCF_048418815.1_sHemAka1.3/	GCF_048418815.1

GCF_021869965.1_sRhiTyp1.1_genomic.gff	*Rhincodon typus*	https://ftp.ncbi.nlm.nih.gov/genomes/all/GCF/021/869/965/GCF_021869965.1_sRhiTyp1.1/	GCF_021869965.1
GCF_048565385.1_mSaiBol1.pri_genomic.gff	*Saimiri boliviensis*	https://ftp.ncbi.nlm.nih.gov/genomes/all/GCF/048/565/385/GCF_048565385.1_mSaiBol1.pri/	GCF_048565385.1
GCF_048593235.1_ASM4859323v1_genomic.gff	*Sminthopsis crassicaudata*	https://ftp.ncbi.nlm.nih.gov/genomes/all/GCF/048/593/235/GCF_048593235.1_ASM4859323v1/	GCF_048593235.1
GCF_049306965.1_GRCz12tu_genomic.gff	*Danio rerio*	https://ftp.ncbi.nlm.nih.gov/genomes/all/GCF/049/306/965/GCF_049306965.1_GRCz12tu/	GCF_049306965.1
GCF_040954835.1_fLepOcu1.hap2_genomic.gff	*Lepisosteus oculatus*	https://ftp.ncbi.nlm.nih.gov/genomes/all/GCF/040/954/835/GCF_040954835.1_fLepOcu1.hap2/	GCF_040954835.1
GCF_049350105.2_T2T-MMU8v2.0_genomic.gff	*Macaca mulatta*	https://ftp.ncbi.nlm.nih.gov/genomes/all/GCF/049/350/105/GCF_049350105.2_T2T-MMU8v2.0/	GCF_049350105.2
GCF_905171775.1_aRanTem1.1_genomic.gff	*Rana temporaria*	https://ftp.ncbi.nlm.nih.gov/genomes/all/GCF/905/171/775/GCF_905171775.1_aRanTem1.1/	GCF_905171775.1
GCF_963506605.1_rZooViv1.1_genomic.gff	*Zootica vivipara*	https://ftp.ncbi.nlm.nih.gov/genomes/all/GCF/963/506/605/GCF_963506605.1_rZooViv1.1/	GCF_963506605.1

GCF_009769535.1_rThaEle1.pri_genomic.gff	*Thamnophis elegans*	https://ftp.ncbi.nlm.nih.gov/genomes/all/GCF/009/769/535/GCF_009769535.1_rThaEle1.pri/	GCF_009769535.1
